# Supplementary material for: Medical students’ attitudes toward psychiatry in Indonesia
Source: PLoS One. 2022 Mar 24;17(3):e0265605. doi: 10.1371/journal.pone.0265605 (PMC8947139; doi:10.1371/journal.pone.0265605)
Supplement: Attachment — Submitted filename: PLOS ONE_REV_PONE_D_21_18251.docx [file pone.0265605.s001.docx]

Title: Medical students’ attitude toward psychiatry in Indonesia: a pilot study

Reviewer’s comments:

TITLE: This is not clear why this is being called “a pilot study”. I have not found any explanation of this while reading through the paper

ABSTRACT: The result segment of the abstract is poorly written. There has been some repetitions and the results were not succinctly present.

INTRODUCTION:

- Several statements were identified that were not provided with in-text citations. The authors should revisit the introduction. For example, line 59-61, page 3 states, “Factors influencing this stigma among the society may be related to the lack of knowledge and information regarding the course of mental illness and its treatment” does not have any citation.
- Some portions of the introduction require rephrasing. For instance, line 72-75, page 4 states, “The media, culture, personality, and even history played part in the formed images of psychiatry. Another factor is the influences of training for medical students. Doctors usually have criticisms of how psychiatry lacks on science and evidence-based theory, as well as demands too much emotion, and criticism of how psychiatrists are not apt to be role models.” This seems unclear and complicated. The authors should try to make it easily understandable. Another sentence in the same page (line 81-82) is ambiguous.

MATERIALS AND METHODS:

- This is not clear why 2^nd^ and 3^rd^ year students were excluded
- The data collection process was not explained well
- There was no section that explained the sample size of the study

RESULTS:

- The socio-demographic characteristics seem incomplete
- In line 128-129 this was mentioned that the mean age of both categories was 21.29 with the same standard deviation. I am not sure whether this is correct or not
- Table 2 and Table 3 could be merged, and again to limit the table size the pre-psychiatry rotation group and post-psychiatry rotation groups can be analysed.
- The differences between pre-rotation and post-rotation groups could be provided as well
- In some cases, there was repetition of findings in narratives and table. This should be avoided

DISCUSSION:

- Discussion is well written

OVERALL COMMENTS:

- This paper is interesting but needs quite a lot of revision. The authors should think how the data could be presented in a more interesting way.
